# A bioinformatic approach to investigate placental and fetal gene expression during diabetic stress in pregnancy

**DOI:** 10.1007/s40203-026-00778-2

**Published:** 2026-10-23

**Authors:** Emily Larson, Aman Ullah

**Affiliations:** 1 School of Systems Biology, George Mason University, 4400 University Drive, Fairfax, VA USA; 2 Interdisciplinary Program in Neuroscience, George Mason University, Fairfax, VA 22030 USA

**Keywords:** Bioinformatics, Diabetic pregnancy, GEO, Placental gene expression, Differentially expressed genes (DEGs), Maternal–fetal interface, Pathway analysis

## Abstract

Maternal diabetes disturbs the intrauterine environment and poses significant risk to fetal development, yet the molecular responses of placental and fetal tissues to diabetic stress remain incompletely characterized. We applied a comparative bioinformatic framework to two publicly available microarray datasets representing placental/decidua and fetal tissue collected at embryonic day 10.5. Both datasets were processed using RMA normalization, differential gene expression analysis, principal component analysis, functional enrichment analysis, and Pathview pathway visualization in R. Placental/decidual exhibited a comparatively constrained differential-expression profile with only two probes reaching BH-adjusted p < 0.05 and one probe meeting both BH-adjusted p < 0.05 and |log2FC| > 1. Rank-based GSEA across the complete gene-ranking distribution was used to identify coordinated pathway-level changes. This analysis identified enrichment of gene programs involving ribosome biogenesis, RNA processing and splicing, cell-cycle and DNA-replication processes, and oxidative phosphorylation. In contrast, fetal tissue exhibited substantially broader differential expression with 2,306 probes meeting both BH-adjusted p < 0.05 and |log2FC| > 1. KEGG over-representation analysis identified 62 significantly enriched pathways, including pathways associated with axon guidance, Wnt signaling, focal adhesion, MAPK signaling, and PI3K-AKT signaling. Pathview mapping further demonstrated a broader distribution of transcript-level alterations across fetal signaling components compared with the comparatively localized changes observed in placental/decidual tissue. These findings demonstrate distinct transcriptional responses between placental/decidual and fetal tissue under diabetic stress. The comparatively constrained placental/decidual differential-expression profile together with broader fetal transcriptional alterations is consistent with a model in which the maternal-fetal interface may modulate responses to diabetic stress. Collectively, this comparative analysis identified coordinated molecular processes associated with diabetic pregnancy and provides a framework for early biomarker development in diabetic pregnancies.

**Supplementary Information:**

The online version contains supplementary material available at https://doi.org/10.1007/s40203-026-00778-2.

## Introduction

The placenta, often referred to as the “forgotten organ,” plays a critical role in maintaining maternal health and supporting fetal development throughout pregnancy (Maltepe and Fisher 2015). Although the placenta continues to mature throughout pregnancy, it is initially formed when the trophoblast layer of the blastocyst implants into the maternal endometrium (Herrick et al. ). As the primary interface between the maternal and fetal environments, the placenta regulates nutrient and oxygen exchange while functioning as an endocrine, metabolic, vascular, and immunological organ that coordinates maternal-fetal communication (Maltepe and Fisher 2015, Herrick et al. , O’Brien and Wang 2023). Consequently, impaired placental function or placental insufficiency can negatively affect fetal development and increase the risk of adverse pregnancy outcomes (Cleveland ).

The Developmental Origins of Health and Disease (DOHaD) hypothesis proposes that environmental exposures during fetal development in utero may influence long term health outcomes later in life (Wadhwa et al. 2009). Maternal diseases such as diabetes can influence fetal development through multiple interconnected biological mechanisms. These mechanisms include alterations in placental structure and function, direct effects on fetal developmental programming and gene expression, and changes in the maternal metabolic and inflammatory environment. Among these interconnected mechanisms, the placenta serves as the principal mediator of maternal-fetal communication, integrating maternal physiological signals and regulating the fetal environment. Accordingly, alterations in placental gene expression and function can substantially modify the intrauterine environment and influence fetal development (O’Brien and Wang 2023). Moreover, recent studies have linked placental function to the development of metabolic disorders, emphasizing the placenta’s role in shaping future disease risk (Salbaum et al. 2011).

Although the exact causes of placental insufficiency are not fully understood and are often multifactorial, several maternal health conditions may contribute to its development. Factors such as maternal stress, poor nutrition, and obesity have been associated with impaired placental function. In addition, broader social stresses such as housing and food insecurity, poverty, and environmental exposure can also contribute to poor placental health and pregnancy outcomes (Thornburg et al. 2020). Many aspects of placental health are associated with maternal lifestyle factors, however genetic variations also play an important role in placental function (Wang et al. 2022). Understanding the complex mechanisms underlying placental insufficiency is essential, as impaired placental health can have significant consequences for both the fetus and the mother. Poor placental function may serve as an early indicator of long-term health risks for the developing fetus, potentially affecting intrauterine growth and future health outcomes. For the mother, these complications during pregnancy and birth can lead to increased risks of cardiovascular disease, chronic hypertension, and stroke later in life (Thornburg et al. 2020). Therefore, maintaining optimal placental health is pivotal for supporting a healthy pregnancy and promoting long-term health for both the mother and child.

The scientific approach to human genome exploration has evolved significantly over the past several decades. Prior to the Human Genome Project (HGP), genome analysis relied on earlier sequencing methods such as Maxam-Gilbert sequencing, Sanger sequencing, and polymerase chain reaction-based approaches (Eren et al. 2022). The HGP represented a major breakthrough in genomic research, utilizing a hierarchical shotgun strategy for large scale sequencing and significantly advancing the study of gene expression (Waterston et al. 2002). In parallel with these developments, microarray technology enabled the simultaneous measurement of thousands of transcripts and became an important platform for genome-wide gene expression profiling. More recently, next-generation sequencing technologies, including RNA sequencing (RNA-seq), have expanded transcriptomic analysis by enabling genome-wide quantification of RNA transcripts (Behjati and Tarpey 2013, Wang et al. 2009). Although RNA-seq is now widely used in transcriptomic research, microarray datasets remain valuable for bioinformatic investigations because of the large number of well-characterized studies deposited in public repositories such as the Gene Expression Omnibus (National ). Established microarray platforms, including the Affymetrix Mouse Genome 430 2.0 Array, provide standardized probe-level measurements that can be reanalyzed using modern normalization, differential expression, and pathway-enrichment methods. In the present study, we applied a bioinformatic workflow in R and RStudio to publicly available Affymetrix microarray datasets to investigate placental/decidual and fetal gene-expression responses to maternal diabetic stress.

## Methods

The Gene Expression Omnibus (GEO) is a public repository overseen by the National Center for Biotechnology Information (NCBI) (National ). Through GEO, a large number of datasets are publicly available and can be filtered to studies relevant to a specific research question. For our study, we searched the GEO dataset browser to filter to our particular interest the keywords “placenta” and “diabetic” (National ). The placental/decidua dataset was identified as accession number GSE28277 (*n* = 10 samples). Microarray data were generated using Affymetrix Mouse Genome 430 2.0 Array. Control sample IDs were labeled GSM699798, GSM699799, GSM699800, GSM699801, and GSM699802 and diabetic sample IDs were labeled as GSM6990803, GSM699804, GSM699805, GSM699806, and GSM699807. The fetal/embryonic tissue dataset was identified as accession number GSE41095 and consisted of six samples including three control samples (GSM1008501-GSM1008503) and three diabetic samples (GSM1008504-GSM1008506). The same Affymetrix Mouse Genome 430 2.0 Array platform was used for gene expression profiling.

Both datasets were publicly deposited by Salbaum et al. in association with a publication examining gene expression effects in diabetic pregnancies in *Mus musculus* (Salbaum et al. 2011). The researchers employed a streptozotocin (STZ)–induced diabetic mouse model to dams (mother mice) to examine gene expression data at day 10.5 of embryonic development (E10.5). STZ is a pharmaceutical drug that destroys pancreatic β-cells and is utilized to induce maternal hyperglycemia during pregnancy (Angelis et al. 2009). The maternal hyperglycemia promotes a hyperglycemic intrauterine environment characterized by elevated glucose, oxidative stress, and inflammatory cytokines (Boardley et al. 2025). RNA was extracted from whole placental tissue which included both maternal deciduae, derived from the uterine lining, and the fetal components containing compositions of trophoblast-derived layers. The samples were homogenized, where both the maternal decidua and fetal compositions were blended at extraction (Salbaum et al. 2011). Together, these datasets allow for the evaluation of the diabetic intrauterine environment across two biologically linked, though distinct, sample types which capture tissue-level effects at the maternal-fetal interface.

### Statistical Analyses

#### Analyses for Placental/Decidua Tissue

In this study, R (v4.4.2) was used for all analyses, with RStudio serving as the integrated development environment. The microarray data were processed and analyzed using established R/Bioconductor packages. The raw expression profiling file was retrieved using the getGEOSuppFiles() function from the GEOquery package (Davis and Meltzer 2007). Before beginning differential gene expression analysis, the raw data were normalized through Robust Multi-array Average (RMA) normalization utilizing the rma() function, and expression values were extracted utilizing the exprs() function-both from the affy package. Following RMA normalization, the samples were grouped according to their experimental condition (control or diabetic).

Preprocessing ensured data quality prior to downstream analysis. Two boxplots were created in R to display the samples prior to normalization and following RMA normalization. The box-and-whisker plots were created utilizing the boxplot() function from the base graphics package of R. An MA plot was created utilizing the plotMA() function from the *limma* package in R (Matthew et al. ). The MA plot illustration allows for both the differentially expressed genes within the statistically significant threshold and those genes that fall outside the threshold to be denoted as dots on the plot. Quality control measures such as the box-and-whisker plots, exploratory visualizations such as MA plots were created during the data normalization and are represented in Supplementary Figure S1-S3.

For the differential gene expression analysis, a Principal Component Analysis (PCA) was produced using the prcomp() function from base R. A PCA was performed to visualize the sample clustering and variance within the diabetic and control placental samples. The variances and clustering are based on gene expression profiles and assist in a clearer understanding of the separation between samples and assessing overall variance structure. Along with dimensional visualizations, the identification of outliers is another useful output of a PCA.

Following the differential gene expression analysis, a volcano plot was generated using the EnhancedVolcano() function from the EnhancedVolcano package to display the relationship between log_2_ fold change (FC) and -log_10_ of the Benjamini-Hochberg (BH)-adjusted *p*-values for the genes of interest. Genes meeting the predefined thresholds (BH-adjusted *p*-value < 0.05 and |log_2_FC| > 1) were considered significantly differentially expressed.

Differential gene expression analysis was performed using the *limma* package in R. A heatmap was generated utilizing the pheatmap() function from the pheatmap package which allowed for a visualization of the differentially expressed genes (DEGs) and statistical significances (Gu and Hübschmann 2022). Expression patterns of the top 15 ranked genes were visualized using a heatmap, and hierarchical clustering was included in the related genes across all samples. Within a heatmap, similar expression trends are visualized in both control and experimental (placental) samples. The genes depicted in our heatmap represented the top ranked differentially expressed genes from the study with BH-adjusted *p*-value < 0.05 and |log_2_FC| > 1 as well as Z-score normalized expression values across genes. Additionally, a dendrogram produced in pheatmap() allows for hierarchical clustering to further depict relationships between the top differentially expressed genes.

Functional enrichment analyses were conducted using the clusterProfiler package in R (Yu et al. 2012). Because only one transcript in the placental/decidual dataset (GSE28277) met the combined differential-expression criteria of BH-adjusted *p* < 0.05 and |log_2_FC| > 1, representation analysis based on threshold DEG list was not considered appropriate. Instead, rank-based gene set enrichment analysis (GSEA) was performed using the complete placental/decidual gene list ranked by the *limma* moderated t-statistic. Gene Ontology Biological Process (GO-BP) and Kyoto Encyclopedia of Genes and Genomes (KEGG) gene sets were evaluated using the gseGO() and gseKEGG() functions. For the fetal dataset (GSE41095), KEGG over-representation analysis was performed using genes meeting BH-adjusted *p* < 0.05 and |log_2_FC| > 1 (Xu et al. 2024).

Gene identifiers were mapped to Entrez Gene identifiers using the org.Mm.eg.db organism-specific database for *Mus musculus* which allowed for gene annotation while the mapIds() command carried out ID mapping. KEGG pathway enrichment was generated utilizing the same gene list with the organism code “mmu.” Statistical significance remained consistent at the BH-adjusted *p* < 0.05 threshold. To visualize the results of the KEGG enrichment pathways, a Pathview representation was created utilizing the pathview() command from the pathview package in R (Luo et al. 2017). Pathway diagrams were generated using the complete Entrez-mapped log_2_FC vectors rather than only genes meeting the DEG significance thresholds. MAPK signaling (mmu04010), PI3K-AKT signaling (mmu04151), and neurodegenerative disease (mmu05022) pathways were examined comparatively in placental/decidual and fetal tissues. Pathview figures were interpreted as descriptive visualizations and were not used to determine direct statistical pathway enrichment or activation.

#### Analyses for Fetal Tissue

RStudio was also utilized for the analyses of fetal tissue. Preprocessing was completed through getGEOSuppFiles, rma(), and exprs(). Similar processing for the differential expression was performed with topTable() and pheatmap(). The volcano plot utilized the same command as placental data- EnhancedVolcano(). Preprocessing and quality control visualizations provided in Supplementary Figures (S1-S3).

For the combined analyses across dataset analysis, the top 10 most variable shared genes between placental and fetal datasets were identified and used for joint principal component analysis and hexbin density plot. Pathway mapping was performed for key KEGG pathways including PI3K-AKT (mmu04151), MAPK (mmu04010), and neurodegeneration — multiple diseases (mmu05022). Cross-dataset expression correlation was assessed using Pearson correlation of log_2_FC values via cor() and visualized with ggplot2. Enrichment analyses used mapIds(), enrichGO(), enrichKEGG(), and pathview(), with org.Mm.eg.db maintained for consistent organism annotation. Array-level quality control was assessed using arrayQualityMetrics().

#### Systematic Workflow

**Fig. 1:**
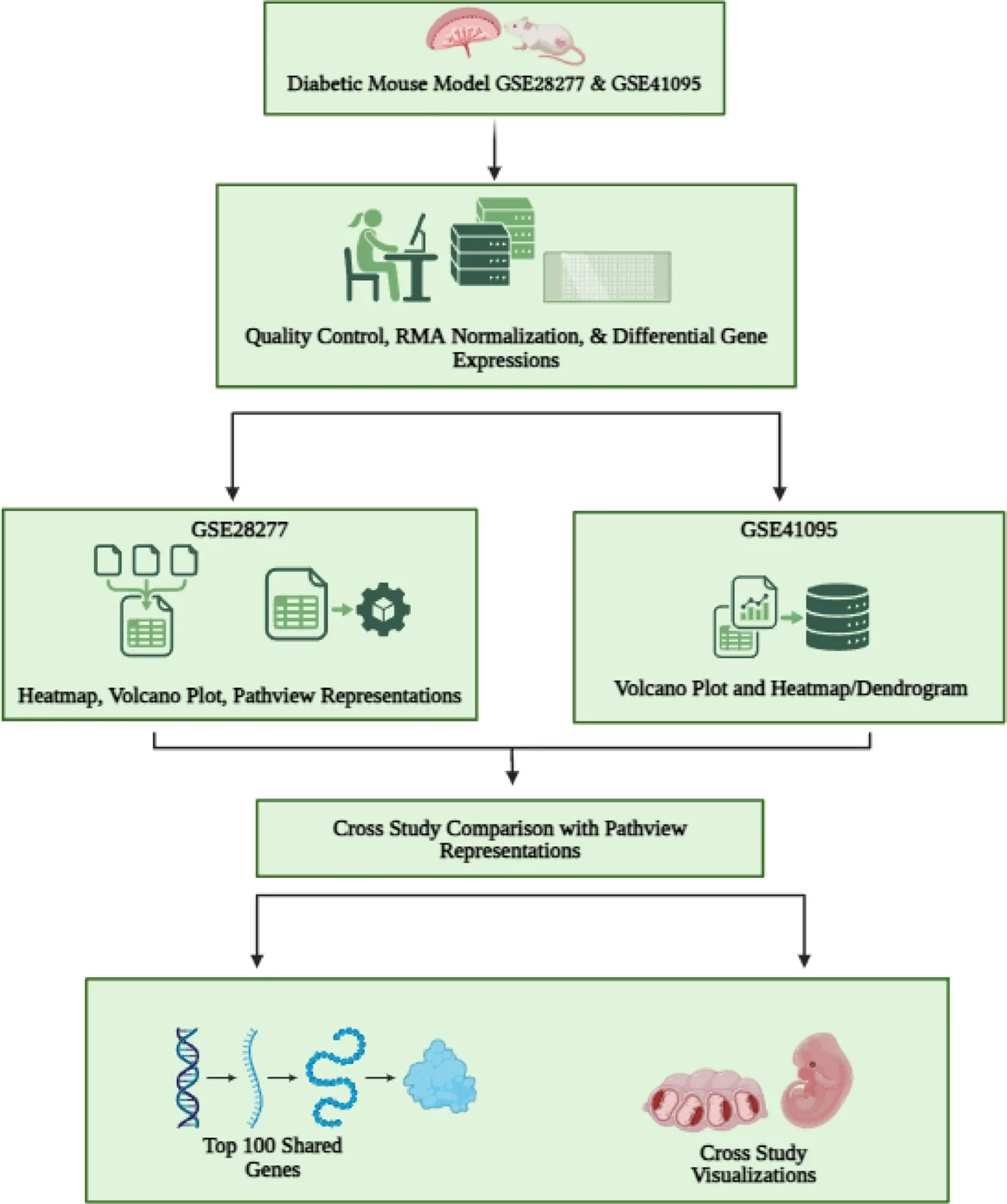
Bioinformatic workflow for cross study analysis of diabetic mouse datasets. Microarray gene expression datasets GSE28277 (placental/decidua) and GSE41095 (fetal tissue) were obtained from the Gene Expression Omnibus (GEO) and were analyzed utilizing a standard bioinformatic workflow. Both raw datasets underwent quality control, Robust Multi-array average (RMA) normalization, and differential gene expression analysis. Downstream analysis consisted of heatmap visualizations, volcano plots, hierarchical clustering, and KEGG pathway mapping using the Pathview package in R. Results from the individual datasets were then integrated to identify transcriptional similarities, followed by cross-study analyses to characterize molecular responses differences within the maternal-fetal interface during diabetic stress in pregnancy. The workflow chart was created through BioRender.com

Systematic workflows can provide transparency and allow for the reproducibility of results. As illustrated in Fig. 1, a systematic workflow was created in BioRender (2025). Microarray-based gene expression profiling was conducted using the mouse model of diabetic pregnancy. The placental dataset was then retrieved using GEOquery in R. Following RMA normalization, differential gene expression analysis was performed, enabling downstream pathway enrichment analysis. Additional analysis in R yielded outcomes including the top 10 significantly enriched KEGG pathways and Pathview visualizations. These results provide insight into the gene expression of placental tissue under diabetic stress conditions. To cross-study the results, we introduced a secondary dataset for fetal tissue. Through analyses, we were able to characterize both distinct and similar differential expressions between the datasets.

## Results

**Fig. 2:**
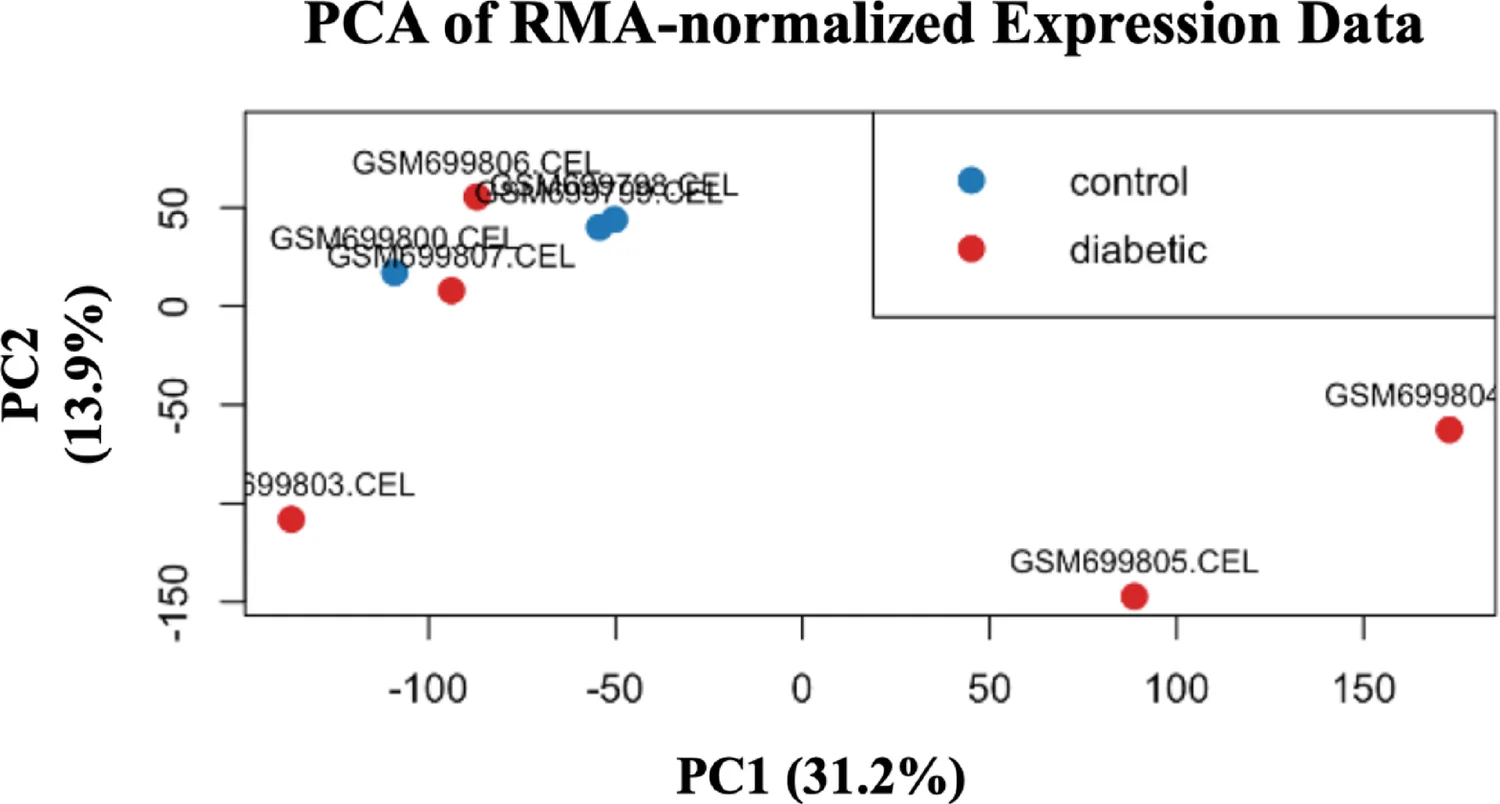
Principal component analysis (PCA) of RMA-normalized gene expression from placental/decidua samples. PC1 and PC2 explain 31.2% and 13.9% of the total variance. Samples are colored according to experimental condition (control, blue; diabetic, red)

PCA was performed on RMA-normalized expression data to assess global variance and sample clustering between control and diabetic groups (Fig. 2). The first two principal components explained 31.2% (PC1) and 13.9% (PC2) of the total variance. A clear separation between control and diabetic samples were observed, indicating distinct transcriptional profiles between conditions. Control samples clustered tightly, particularly along PC2, suggesting a high degree of similarity in gene expression. In contrast, diabetic samples exhibited greater dispersion, with GSM699803, GSM699805, and GSM699804 contributing to increased variability within the group.

**Fig. 3:**
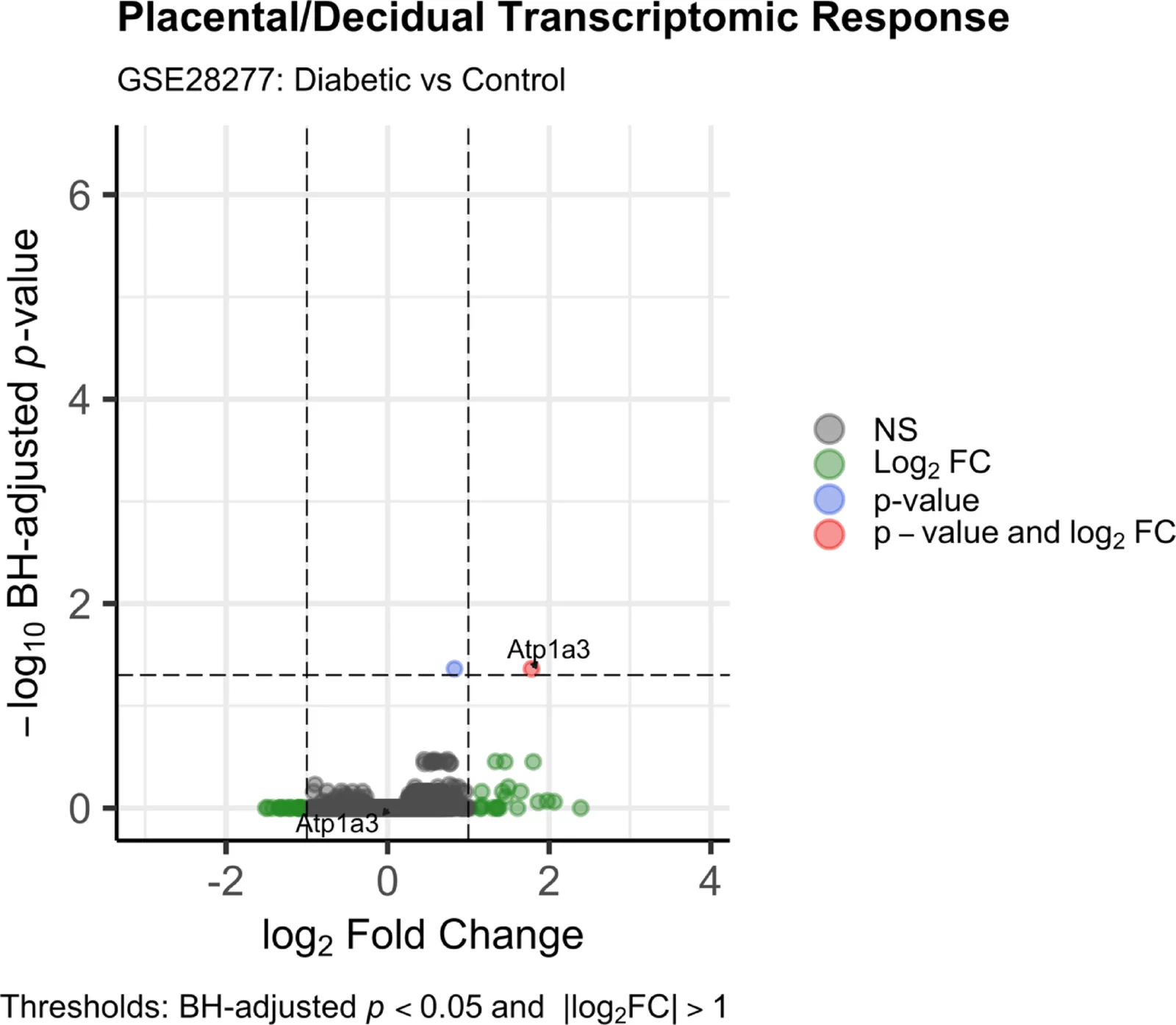
Differential gene expression analysis in placental/decidual tissue under diabetic conditions. Volcano plot displaying log_2_ fold change versus -log_10_ BH-adjusted *p*-value from *limma*-based analysis of RMA-normalized microarray data. Horizontal and vertical dashed lines indicate significance thresholds. Genes meeting both criteria were considered significantly differentially expressed

Following differential gene expression analysis, a volcano plot was generated (Fig. 3). A total of 45,101 probes were analyzed. Genes were evaluated using thresholds of |log_2_FC| > 1 and BH-adjusted *p* < 0.05. The volcano plot displays each probe according to the thresholds with points colored according to significance category: non-significant (gray), meeting only the |log_2_FC| > 1 criterion (green), meeting only the BH-adjusted *p*-value criterion (blue), or meeting both criteria (red).

Differential expression analysis identified two probes with BH-adjusted *p* < 0.05. The probe corresponding to *Atp1a3* exhibited a log_2_ fold change of 1.78 and a BH-adjusted *p*-value of 0.043, meeting both predefined significance criteria. A second probe corresponding to *Rnf182* reached statistical significance (BH-adjusted *p* = 0.043) but exhibited a log_2_ fold change of 0.83 and therefore did not meet the |log_2_FC| > 1 threshold. Thus, only one placental/decidual probe met both predefined criteria for significant differential expression.

**Fig. 4:**
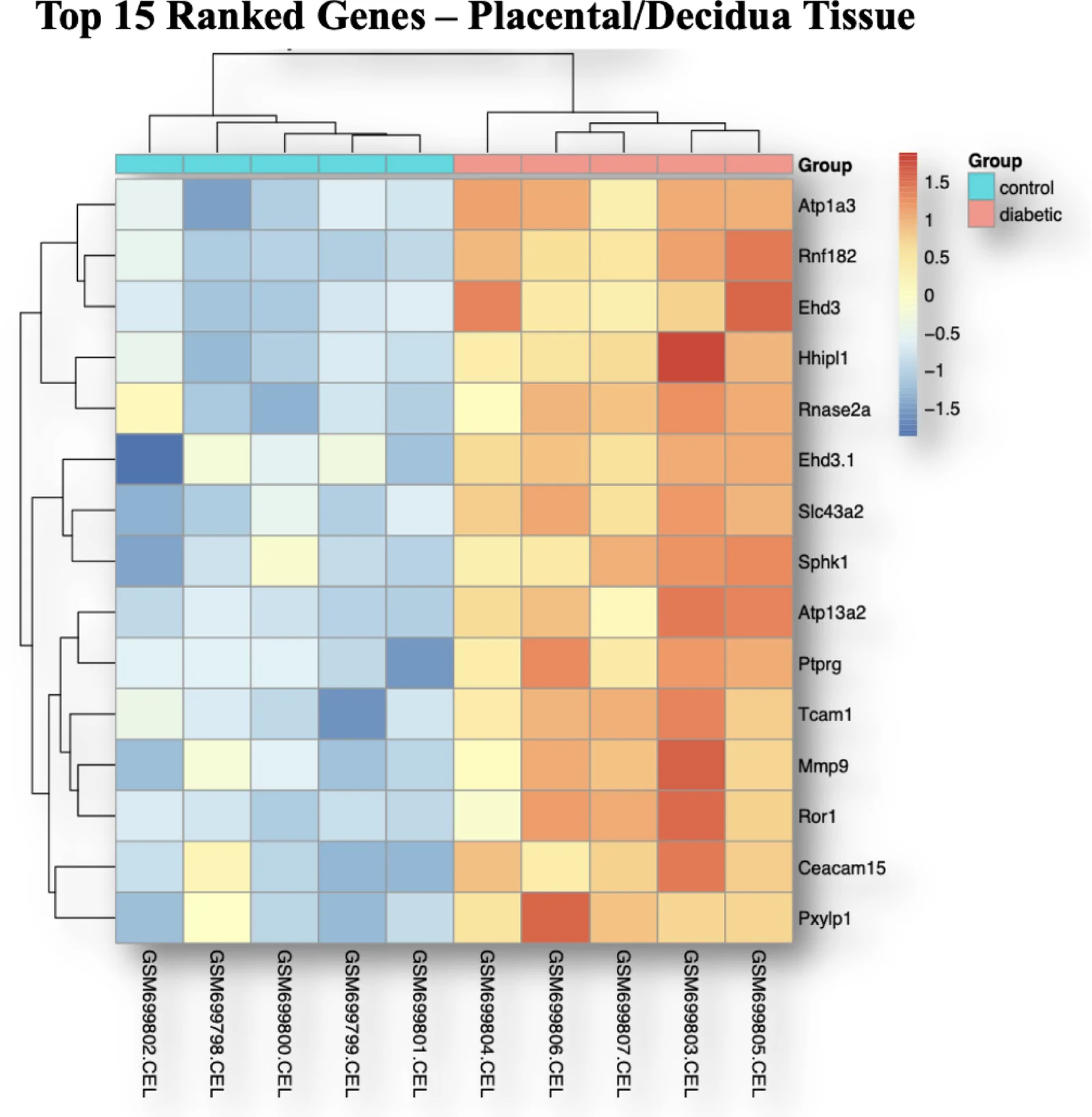
Heatmap of the top 15 ranked genes in placental/decidua tissue based on BH-adjusted *p*-value. RMA-normalized, row-scaled expression values are shown across samples (control vs. diabetic)

To visualize gene expression patterns, a heatmap of the top 15 ranked genes, ordered by BH-adjusted *p*-value, was generated using the pheatmap package (Fig. 4). Expression values were log_2_-transformed and row-scaled, and hierarchical clustering was performed using Euclidean distance. Rows represent genes and columns represent samples (diabetic and control). Color intensity reflects relative expression levels, with red indicating higher expression and blue indicating lower expression relative to the gene-wise mean. The heatmap demonstrates clear separation between diabetic and control samples, consistent with the observed differential expression patterns. Hierarchical clustering along both axes further groups genes and samples with similar expression profiles, revealing coordinated patterns of upregulation and downregulation. Notably, the majority of the top genes exhibit higher expression in diabetic samples relative to controls, supporting a condition-specific transcriptional response.

**Fig. 5:**
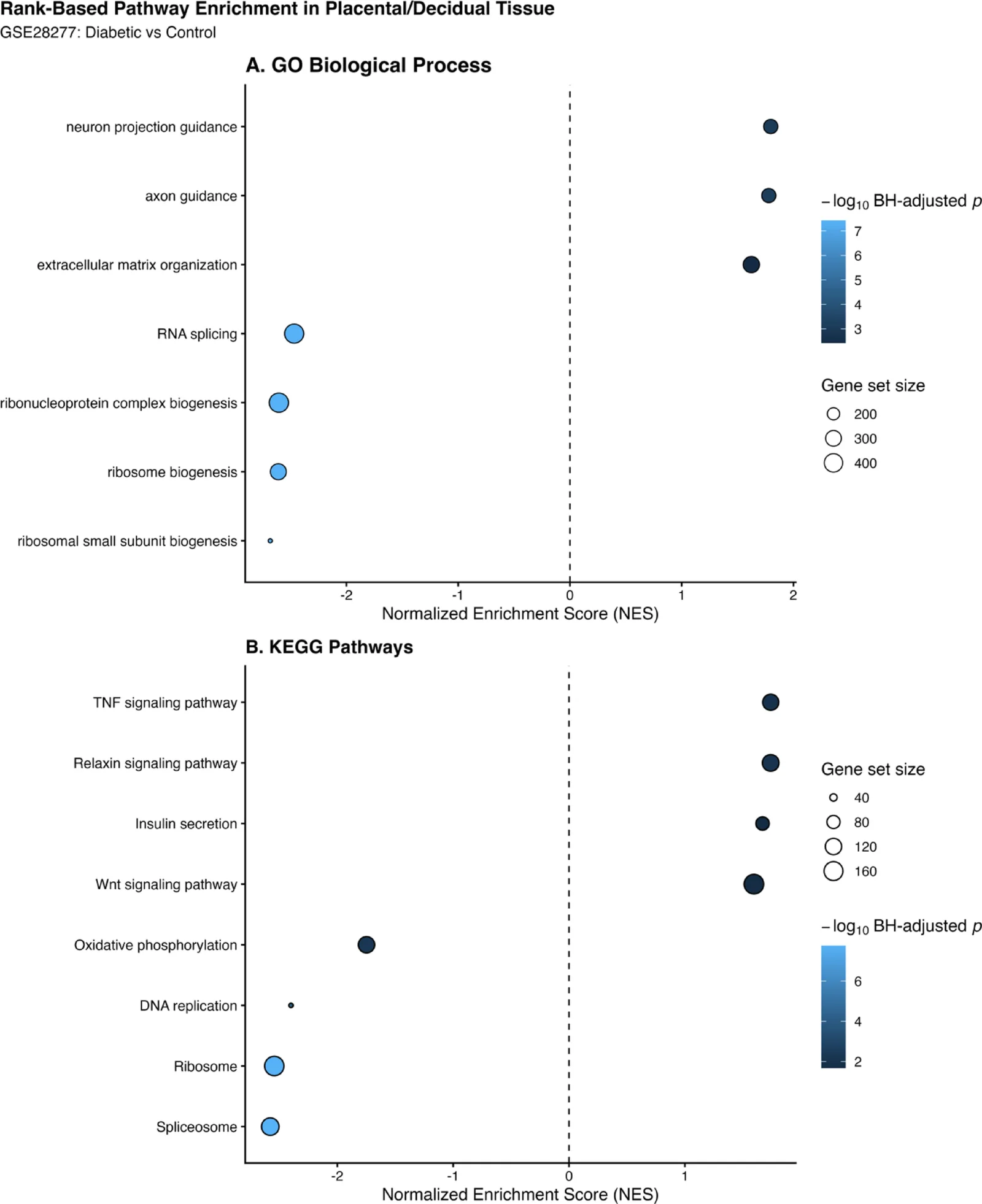
Rank-based gene set enrichment analysis of placental/decidual tissue from diabetic versus control pregnancies (GSE28277). Gene set enrichment analysis (GSEA) was performed using the complete gene list ranked by the *limma* moderated *t*-statistic. (A) Gene Ontology Biological Process (GO-BP) enrichment and (B) Kyoto Encyclopedia of Genes and Genomes (KEGG) pathway enrichment. Normalized enrichment scores (NES) indicate direction and magnitude of enrichment, where positive NES values correspond to genes with higher expression in diabetic pregnancies and negative NES values correspond to genes with lower expression in diabetic pregnancies. Significance was assessed using BH-adjusted p-values

Rank-based gene set enrichment analysis (GSEA) was utilized to complete gene-ranking distribution to assist in identified pathway-level alterations (Fig. 5). GO Biological Process analysis highlighted both positively and negatively enriched processes (Fig. 5A). Ribosomal small subunit biogenesis (NES = -2.68), ribosome biogenesis (NES = -2.61), ribonucleoprotein complex biogenesis (NES = -2.60), RNA splicing (NES = -2.47), rRNA processing (NES = -2.45), and mRNA processing (NES = -2.43) were among the strongest negatively enriched processes. Positively enriched processes included axon guidance, neuron projection guidance, and extracellular matrix organization while negatively enriched processes included RNA splicing, ribosome biogenesis, ribosomal small subunit biogenesis, and ribonucleoprotein complex biogenesis.

Similar pathway-level alterations were identified in KEGG pathways for placental/decidual tissue (Fig. 5B). For KEGG GSEA, negatively enriched pathways were among the highest-ranked significant pathways. Spliceosome (NES = -2.58) and ribosome (NES = -2.54) was among the strongest negative enrichment. Positive enrichment was observed among several pathways. These pathways included TNF signaling, Relaxin signaling, insulin secretion, Wnt signaling. Complete GO and KEGG GSEA results, included gene-set sizes, NES scores, and core enrichment genes are provided in Supplementary Tables S1A and S1B.

**Fig. 6:**
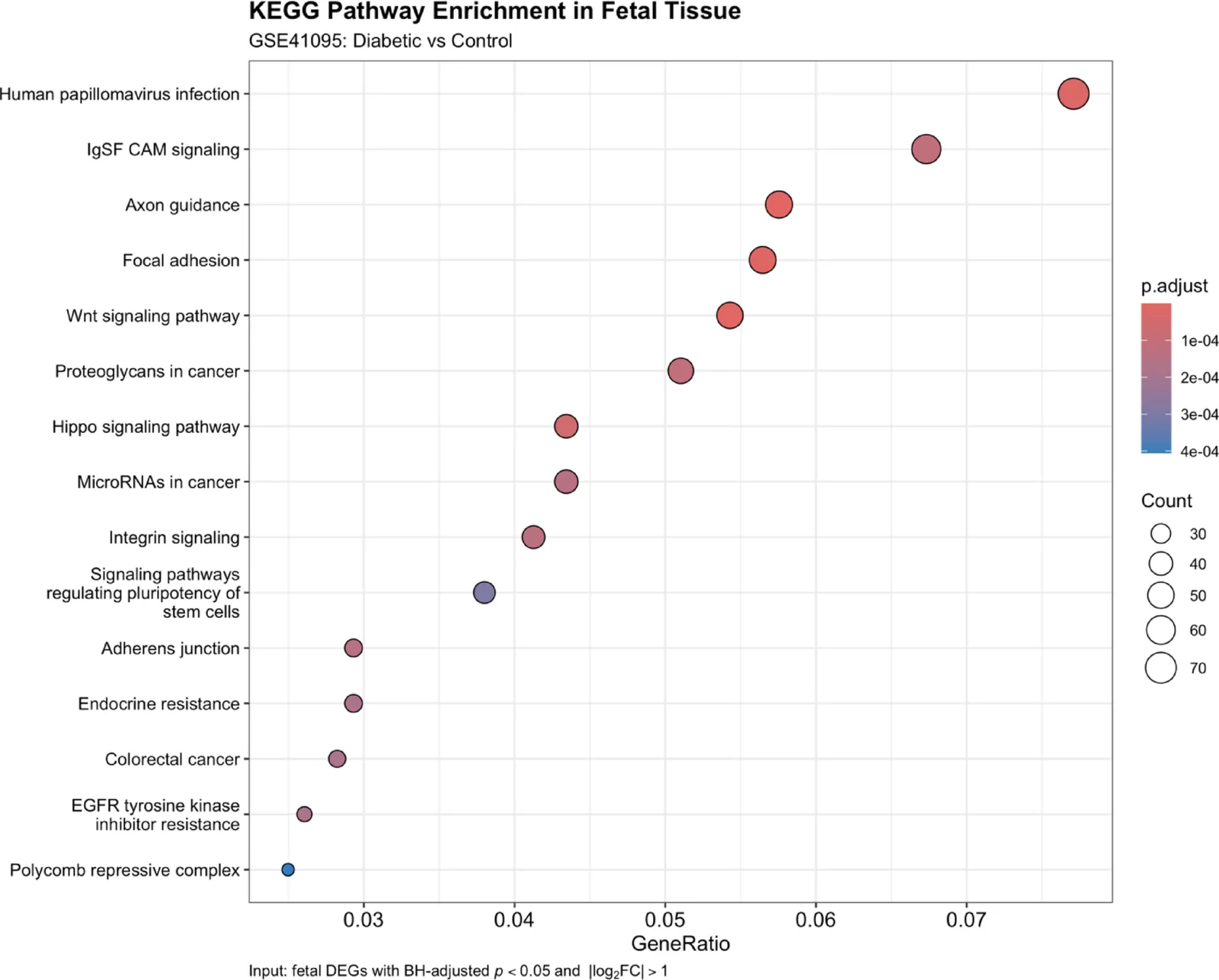
KEGG pathway enrichment analysis of differentially expressed genes in fetal tissue (diabetic vs. control). Dot plot of significantly enriched pathways. The x-axis (GeneRatio) represents the proportion of genes per pathway; dot size indicates gene count and color reflects adjusted *p*-value

Over-representation KEGG analysis of the fetal tissue dataset was performed using the genes meeting the established BH-adjusted *p* < 0.05 and |log_2_FC| > 1. From the analysis, 62 KEGG pathways were significantly enriched at the BH-adjusted *p* < 0.05. Of 2,306 probes meeting both criteria, 2,195 had Entrez Gene annotations which corresponded to 1,838 unique Entrez Gene identifiers. The 1,838 genes were submitted to enrichKEGG with all 21,028 unique Entrez-mapped genes specified as the background universe. The 921 input genes and 7,717 background genes were represented in the KEGG annotation with the predetermined significance threshold of BH-adjusted *p* < 0.05. A complete scope of the enrichment results and the genes contributing to each pathway are provided in Supplementary Table S2. Significantly enriched pathways are visualized in Fig. 6.

Among the most significantly enriched pathways were axon guidance (GeneRatio = 53/921, BH-adjusted *p* = 4.66 × 10^− 8^), focal adhesion (52/921, BH-adjusted *p* = 2.28 × 10^− 6^), and Wnt signaling (50/921, BH-adjusted *p* = 1.61 × 10^− 7^). Several additional pathways were significantly enriched including integrin signaling, endocrine resistance, signaling pathways of regulating pluripotency of stem cells, and adherens junction. Additionally, MAPK signaling (60/921, BH-adjusted *p* = 4.05 × 10^− 4^) and PI3K-Akt signaling (64/921, BH-adjusted *p* = 2.75 × 10^− 3^) were significantly enriched and were examined using pathway-level visualization.

**Fig. 7:**
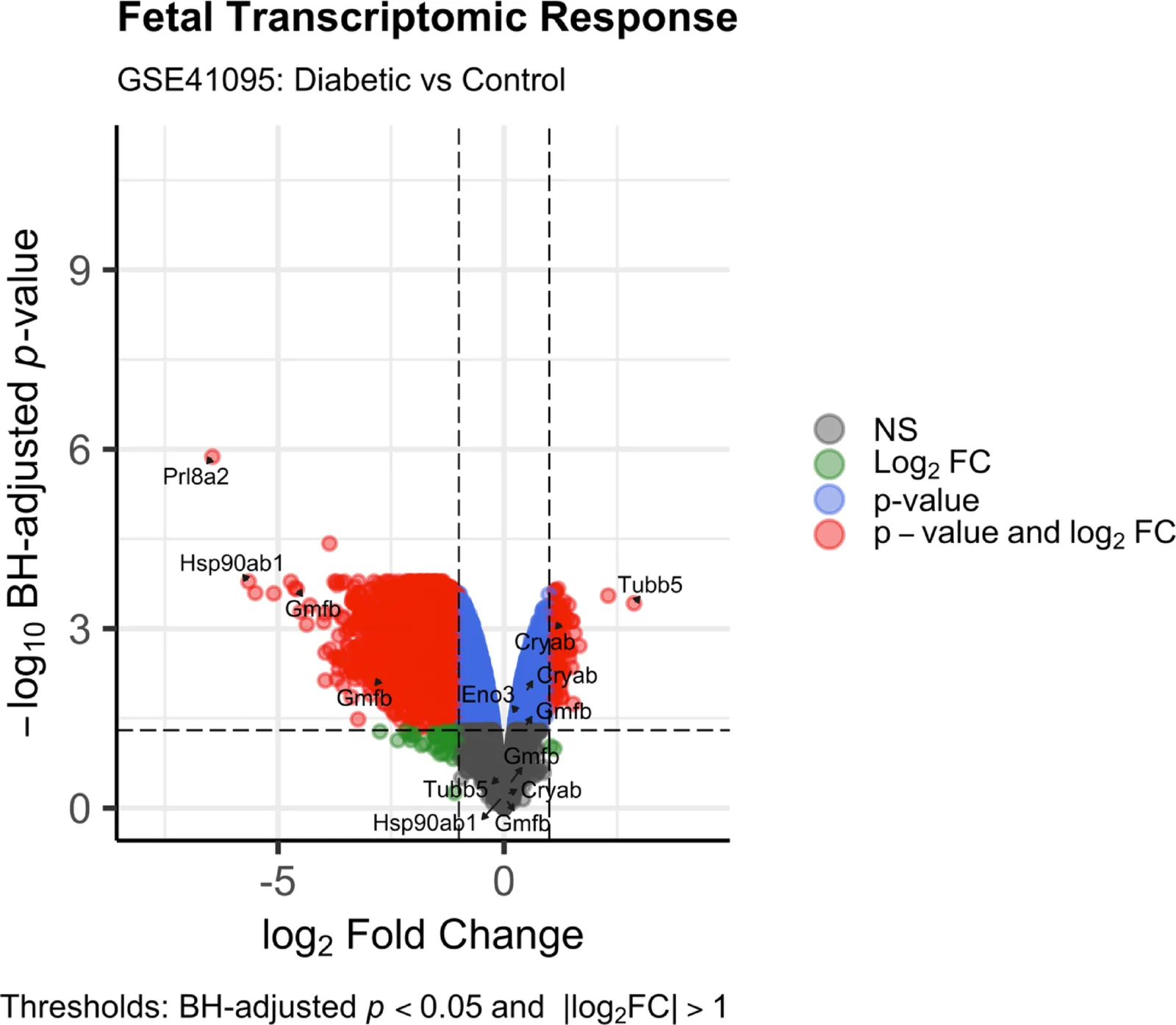
Differential gene expression analysis in fetal tissue under diabetic conditions. **(A)** Volcano plot showing log_2_ fold change versus the -log_10_ BH-adjusted *p*-value from *limma*-based analysis of RMA-normalized microarray data. Genes meeting significant thresholds (|log_2_FC| > 1 and *p* < 0.05) are highlighted and color-coded by category

Differential expression analysis of fetal tissue identified substantial transcriptional alterations between diabetic and control samples (Fig. 7). Applying thresholds of BH-adjusted *p*-value < 0.05 and |log_2_FC| > 1, numerous genes were significantly differentially expressed. A total of 45,101 probes were analyzed with 15,844 probes reaching BH-adjusted *p* < 0.05, and 2,306 probes met both predefined criteria for significant differential expression. A total of 148 probes exhibited positive log_2_ fold changes and 2,158 exhibited negative log_2_ fold changes. *Prl8a2* exhibited the largest negative fold change (log_2_FC = -6.45; BH-adjusted *p* = 1.34 × 10^− 6^). Additional decidual- and trophoblast-associated markers were examined to assess whether differential maternal-interface tissue carry-over could have contributed (Supplementary Table S1C).

**Fig. 8:**
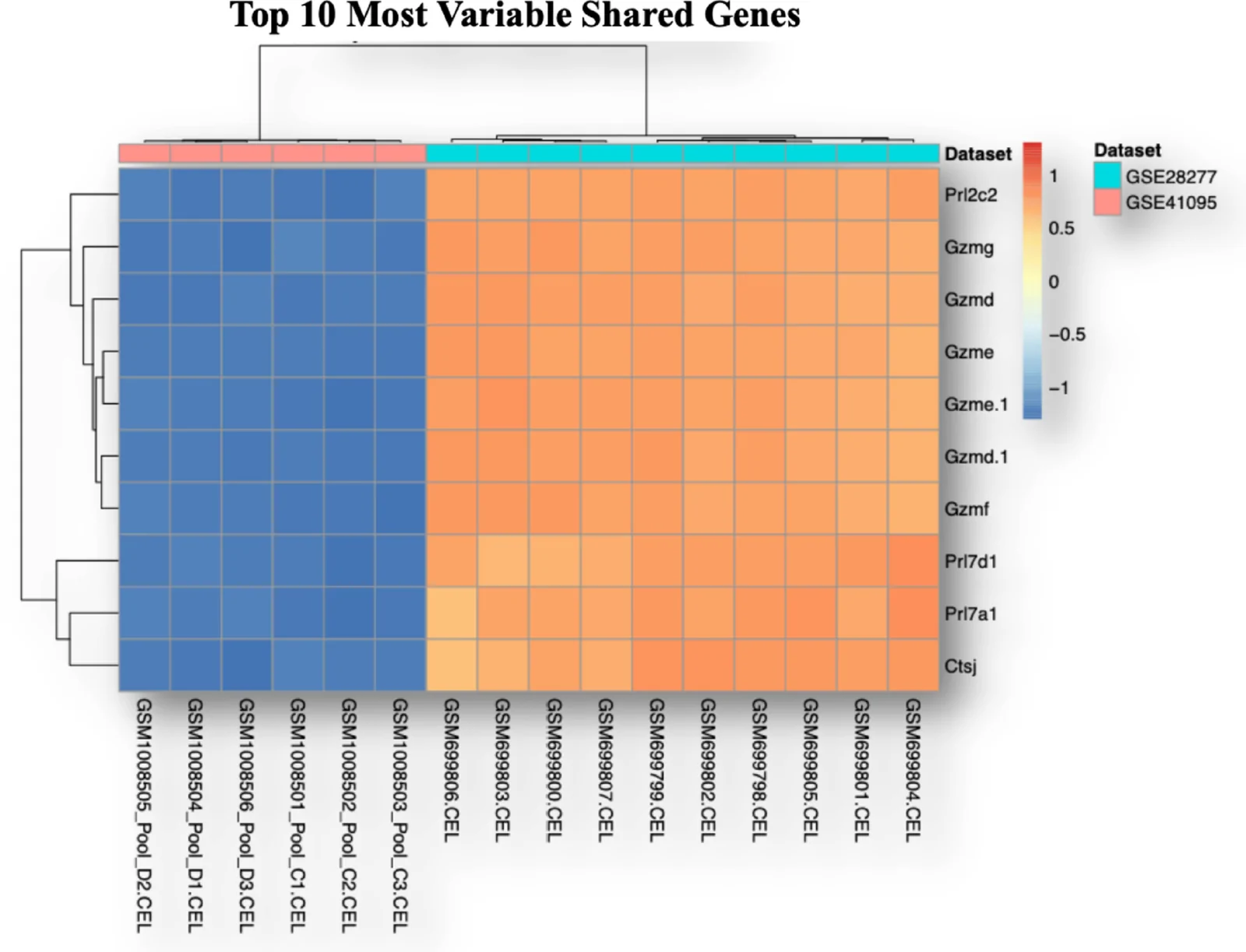
Heatmap of the top 10 variable genes shared between placenta/decidua and fetal tissue. Expression values are RMA-normalized and row-scaled. Rows represent genes and columns represent samples, with annotation dataset origin; warmer colors indicate higher expression levels, whereas cooler colors indicate lower expression levels

A heatmap of the top variable genes shared across datasets revealed distinct clustering patterns between samples (Fig. 8). Hierarchical clustering separated samples into groups with opposing expression profiles, with one cluster showing elevated expression in a subset of samples and corresponding cluster exhibiting reduced expression in the same group. These patterns indicate coordinated transcriptional differences across the shared gene set and support cross-study consistency in expression trends.

Several genes contributed prominently to these patterns, including *Lcn2* (Lipocalin 2), *Kcnq2*, and *Scd1*. Notably, *Lcn2*, an adipokine associated with inflammation and insulin resistance, showed elevated expression consistent with a stress-responsive transcriptional profile. Together, these results suggest that shared genes across datasets capture biologically relevant signals related to metabolic and inflammatory responses under diabetic conditions.

**Fig. 9:**
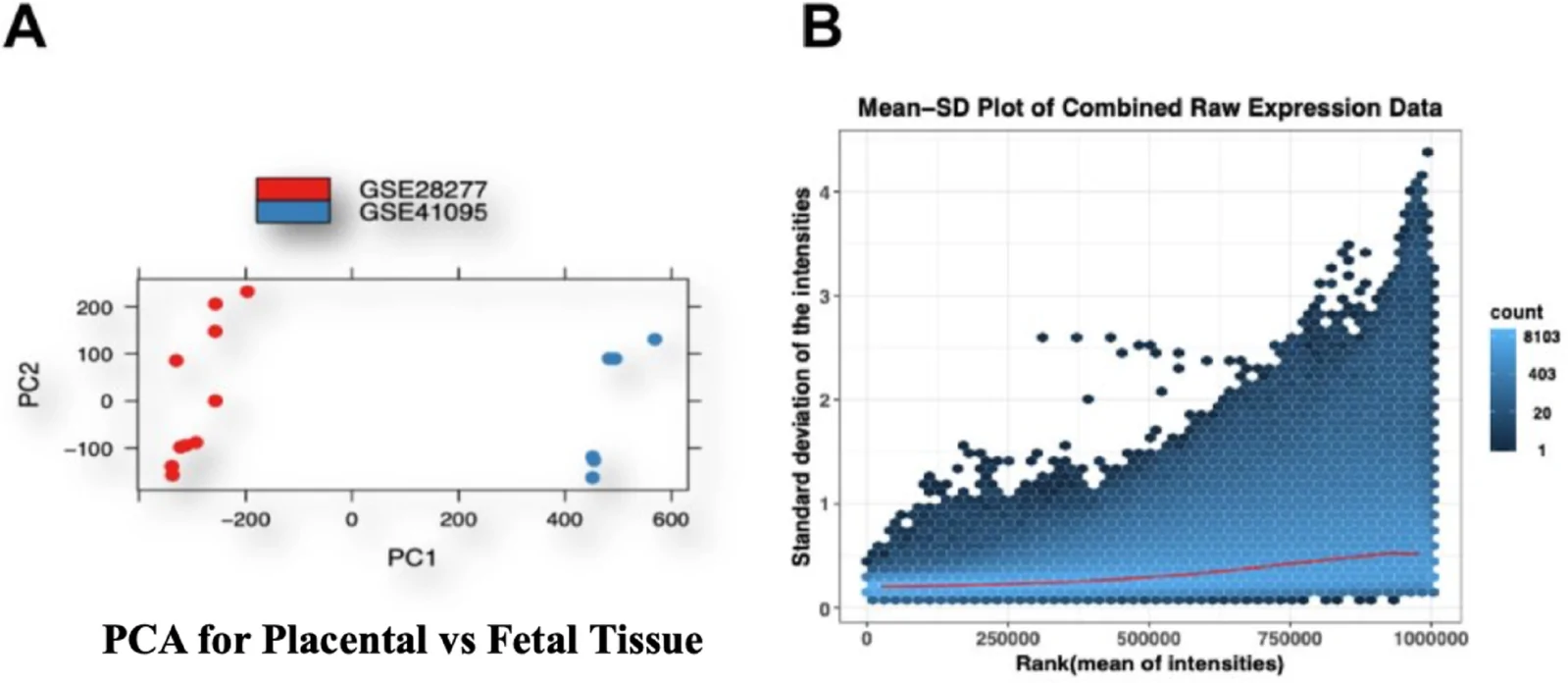
**(A)** Principal component analysis (PCA) of normalized gene expression data from placental/decidua and fetal tissues. Samples are colored by dataset (placental/decidua: red; fetal: blue). **(B)** Relationship between mean probe intensity and standard deviation across arrays for placental/decidua and fetal tissues. Hexbin plot of raw microarray data, with the x-axis showing ranked mean intensity and the y-axis showing standard deviation. Color indicates probe density, and the red line represents the fitted trend

To assess the relationship between placental and fetal datasets, principal component analysis (PCA) was performed on the combined expression data (Fig. 9A). Samples segregated distinctly along PC1, with placental samples (GSE28277) clustering separately from fetal samples (GSE41095), indicating clear separation between the two datasets. Given that the datasets represent biologically distinct tissues from independent studies, this analysis serves as an exploratory visualization of overall transcriptomic differences between placental and fetal tissues. Within each dataset, samples clustered tightly, supporting consistency in expression profiles and overall data quality. Modest dispersion within groups was observed, which may reflect biological variability under diabetic conditions. Collectively, these results demonstrate distinct global transcriptomic profiles between placental and fetal compartments and provide context for the downstream comparative analyses.

To assess variability in probe intensities across arrays, the relationship between mean expression and standard deviation was examined (Fig. 9B). Variability increased with higher mean intensities, consistent with expected mean-variance relationships in raw microarray data. Most probes exhibited low standard deviations, indicating overall technical consistency across arrays. The gradual increase in variability at higher expression levels likely reflects intensity-dependent effects inherent to microarray measurements.

**Fig. 10:**
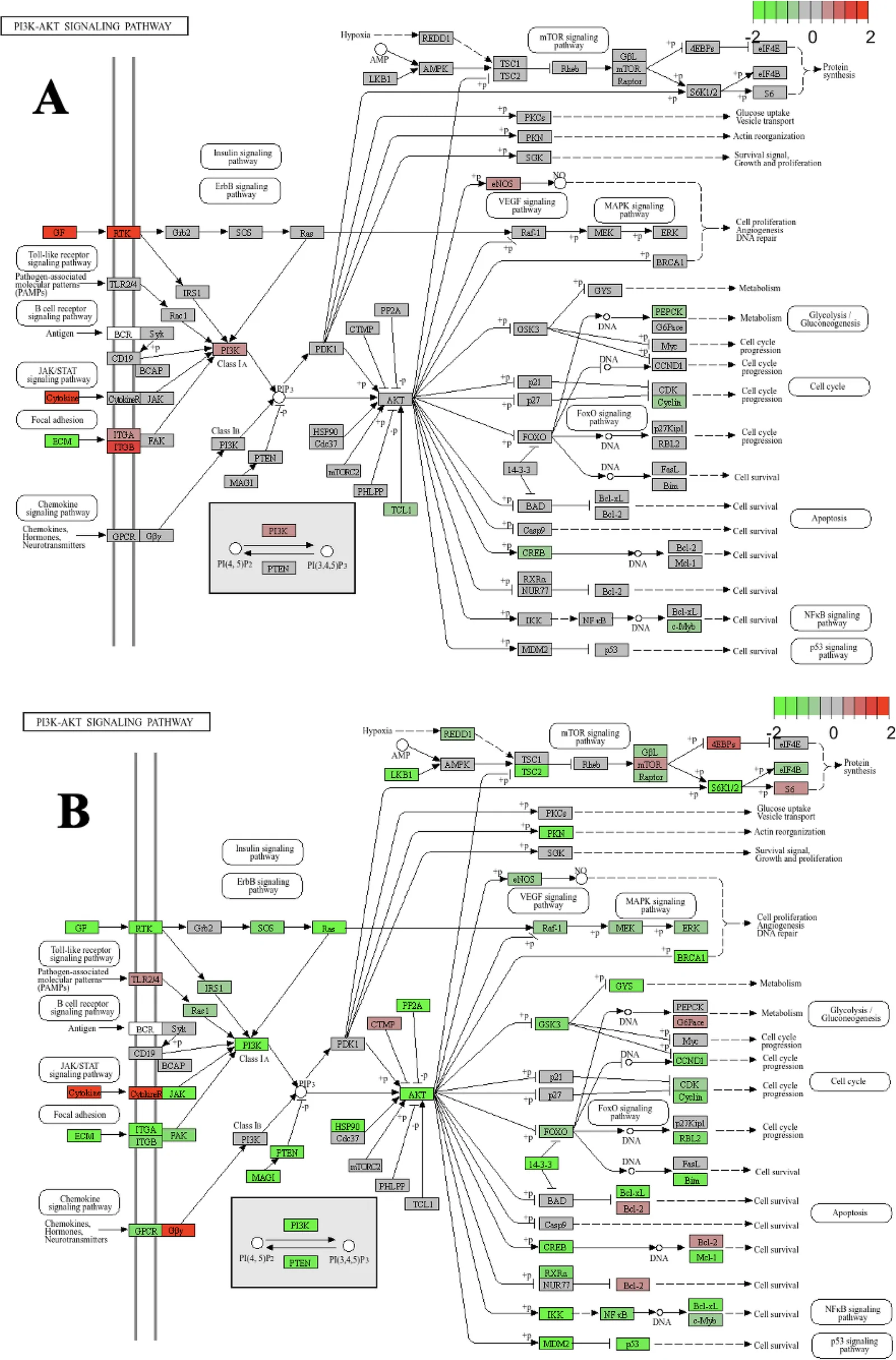
**(A**) KEGG PI3K-AKT signaling (mmu04151) rendered by Pathview for placental/decidua tissue (diabetic vs. control). Differentially expressed genes are mapped and color-coded by log_2_ fold change, with red indicating upregulation and green indicating downregulation. **(B)** KEGG PI3K-AKT signaling pathway (mmu04151) rendered by Pathview for fetal tissue (diabetic vs. control). Differentially expressed genes are mapped and color-coded by log_2_ fold change, with red indicating upregulation and green indicating downregulation

Mapping differential expression onto the PI3K-AKT pathway (mmu04151) revealed marked differences in pathway activity between placental and fetal tissues (Fig. 10). In placental tissue, transcriptional changes were limited, with relatively few pathway nodes exhibiting notable fold changes, indicating a modest perturbation of the PI3K-AKT network.

Fetal tissue showed widespread transcriptional alterations across the pathway, including upstream receptor-associated components, the central PI3K-AKT signaling axis, and downstream effectors involved in mTOR signaling, cell cycle regulation, apoptosis, and metabolic processes. Consistent with the mapped transcript-level alterations, PI3K-AKT signaling was significantly enriched in the corrected fetal KEGG over-representation analysis (GeneRatio = 64/921; BH-adjusted *p* = 2.75 × 10^− 3^).

These results demonstrate a more extensive engagement of the PI3K-AKT pathway in fetal tissue compared to the comparatively attenuated response observed in placental tissue.

**Fig. 11:**
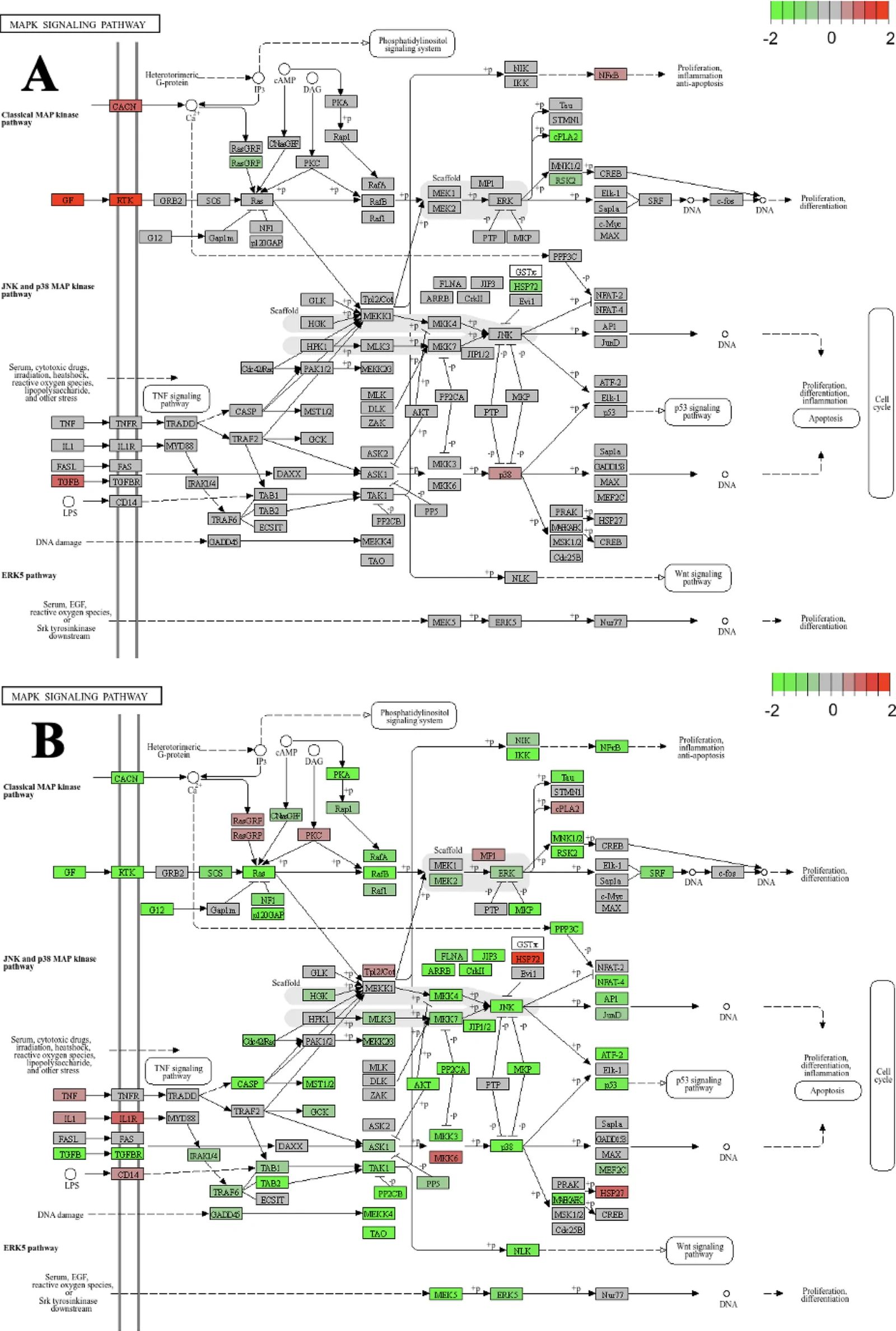
**(A)** KEGG MAPK signaling pathway (mmu04010) rendered by Pathview for placental/decidua tissue (diabetic vs. control). Differentially expressed genes are mapped and color-coded by log_2_ fold change, with red indicating upregulation and green indicating downregulation. **(B)** KEGG MAPK signaling pathway (mmu04010) rendered by Pathview for fetal tissue (diabetic vs. control). Differentially expressed genes are mapped and color-coded by log_2_ fold change, with red indicating upregulation and green indicating downregulation

Mapping differential expression onto the MAPK signaling pathway (mmu04010) revealed comparatively localized transcriptional changes in placental/decidual tissue (Fig. 11A). Most pathway nodes remained near baseline, with only modest upregulation observed in select upstream components including receptor-associated and growth factor-associated nodes. Downstream MAPK cascade element showed minimal expression changes.

In contrast, fetal tissue exhibited widespread transcriptional alterations across the MAPK pathway (Fig. 11B. Alterations were seen across multiple upstream and scaffold components, MAPK cascade intermediates, and downstream stress- and transcription-associated components. Several components exhibited negative log_2_ fold changes. Conversely, stress- and inflammation-associated genes exhibited positive changes. MAPK signaling was significantly enriched in the corrected fetal KEGG over-representation analysis (GeneRatio = 60/921; BH-adjusted *p* = 4.05 × 10^− 4^).

Collectively, these findings indicate differential regulation of MAPK signaling between tissues, with minimal activation in placental tissue and a more extensive, but mixed, transcriptional response in fetal tissue characterized by suppression of core signaling components alongside activation of stress-response pathways.

**Fig. 12:**
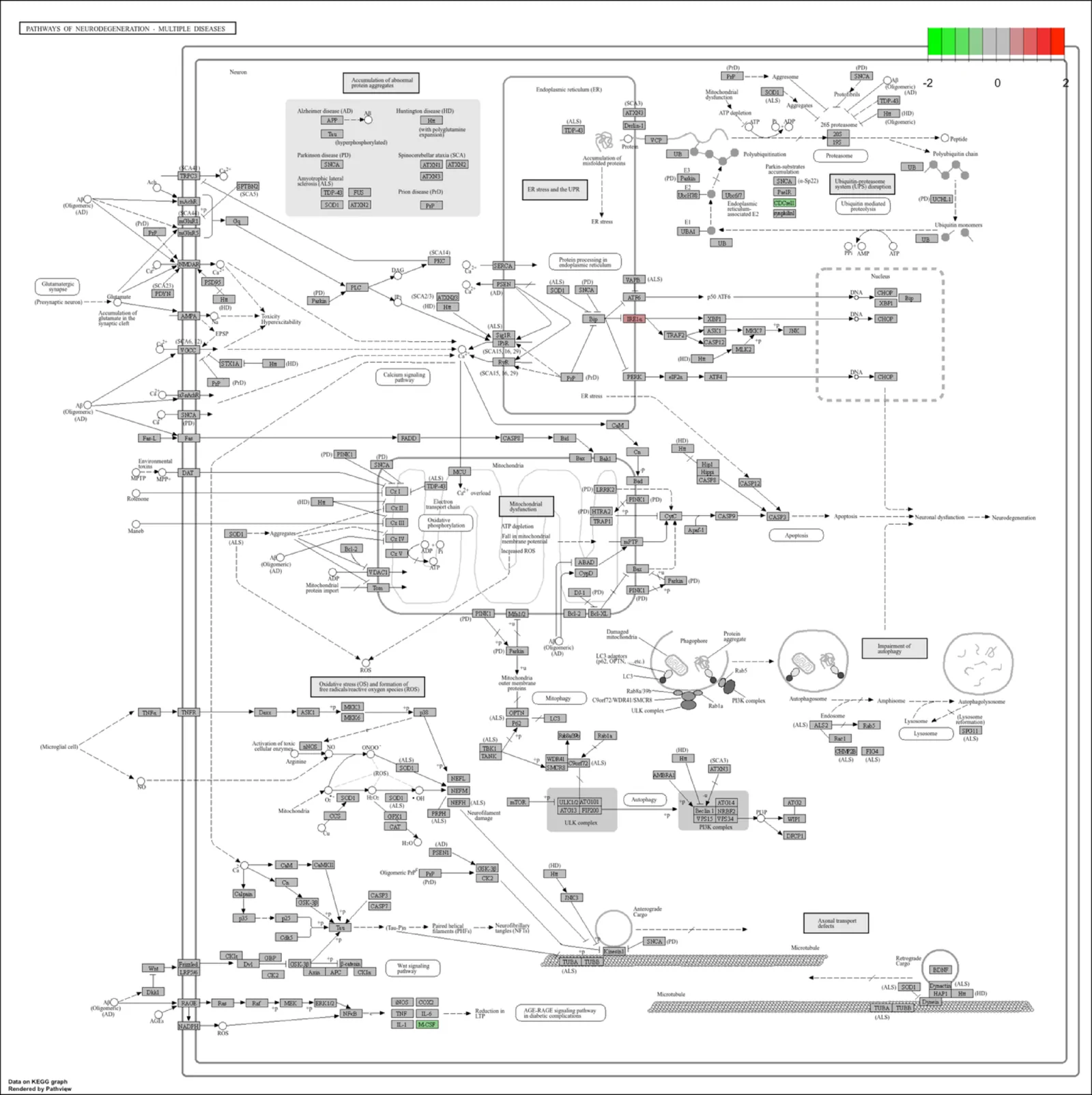
KEGG neurodegeneration - multiple diseases pathway (mmu05022) rendered by Pathview for placenta/decidua tissue (diabetic vs. control). Differentially expressed genes are mapped and color-coded by log_2_ fold change, with red indicating upregulation and green indicating downregulation

**Fig. 13:**
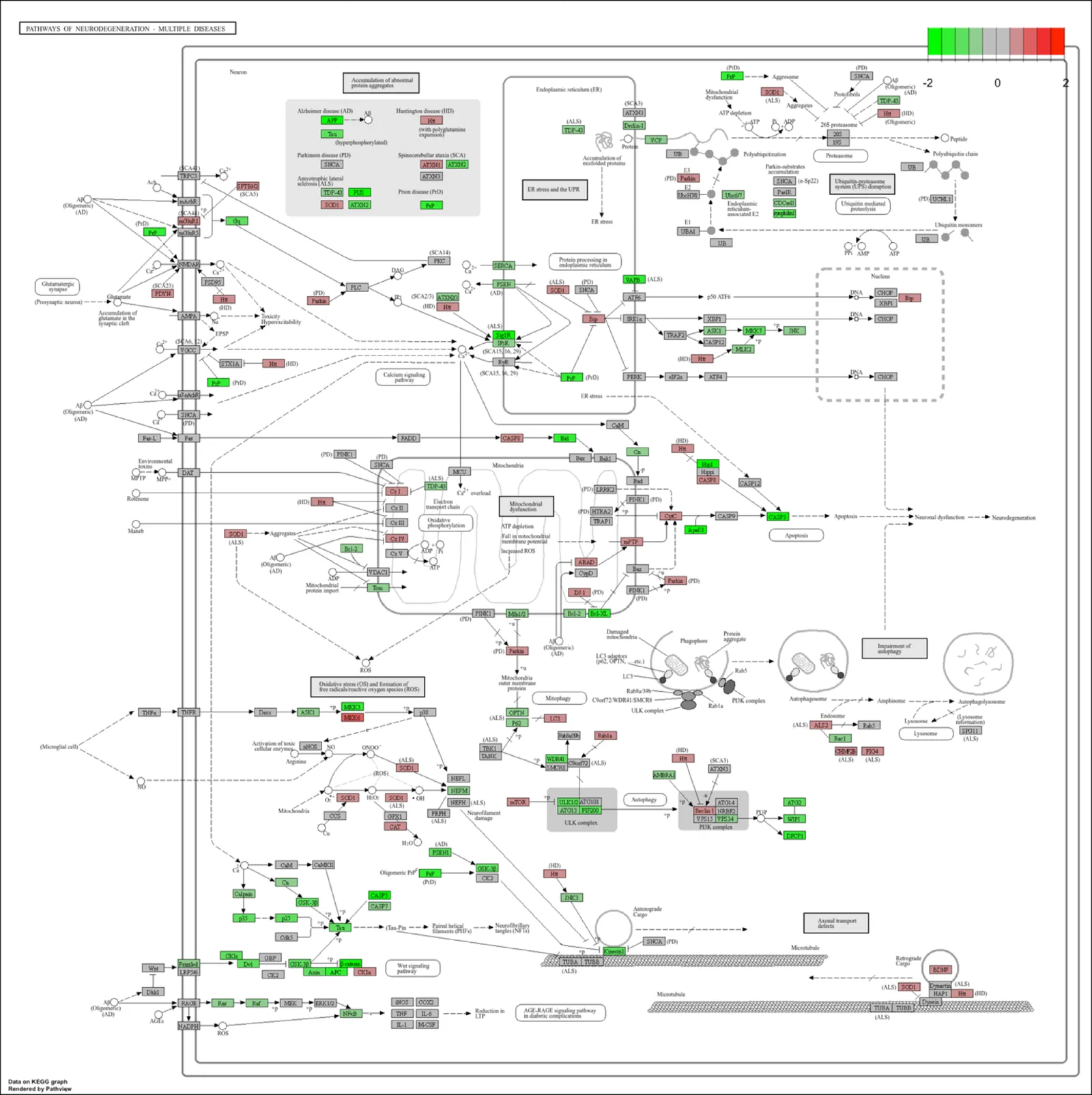
KEGG neurodegeneration - multiple diseases pathway (mmu05022) rendered by Pathview for fetal tissue (diabetic vs. control). Differentially expressed genes are mapped and color-coded by log_2_ fold change, with red indicating upregulation and green indicating downregulation

The KEGG pathway “Pathways of Neurodegeneration – Multiple Diseases” (mmu05022) revealed individual transcriptional patterns between placental and fetal tissues (Figs. 12 and 13). In placental tissue, only a limited number of pathway nodes exhibited notable changes. Several mitochondrial electron transport chain components, including were *NDUFA*,* NDUFB*, and *SDHB*, were moderately downregulated, suggesting mild alterations in mitochondrial function. In contrast, select nodes related to oxidative phosphorylation and protein degradation showed slight upregulation. Overall, transcriptional changes in placental tissue were limited and resembled the subtle perturbations observed in other pathways.

In fetal tissue, more extensive transcriptional alterations were observed across the pathway. Multiple mitochondrial components, including *NDUFS*, cytochrome c oxidase (*COX*) subunits, and ATP synthase genes, were downregulated, consistent with broader disruption of mitochondrial processes. In contrast, genes associated with the ubiquitin-proteasome system and autophagy pathways were upregulated, indicating increased protein turnover and stress response activity. Additionally, apoptosis-related genes, including *CASP3* and *BAX*, exhibited differential expression, alongside changes in neuroinflammatory signaling components.

Together, these results indicate a more pronounced engagement of stress and degeneration-associated pathways in fetal tissue, whereas placental tissue exhibits comparatively limited transcriptional perturbation.

Summary statistics of log_2_ fold changes revealed distinct distributional differences between placental and fetal tissues. In placental tissue, log_2_FC values were tightly centered around zero (median $\:\approx\:$ 0.0015; IQR: -0.077 to 0.079), with a relatively narrow range (-1.51 to 2.39), indicating limited transcriptional deviation. In contrast, fetal tissue exhibited a broader distribution (median $\:\approx\:$ 0.107; IQR: -0.128 to 0.284) and a substantially wider range (-6.45 to 2.87), reflecting greater variability and more extreme gene expression changes.

Notably, the extended negative tail in fetal tissue indicates stronger downregulation in a subset of genes, while the overall wider interquartile range supports increased transcriptional dispersion. These distributional differences are consistent with pathway-level observations, where placental tissue showed relatively subtle transcriptional modulation, whereas fetal tissue exhibited a broader dynamic range of expression changes, corresponding to more pronounced pathway activity.

Across all analyses, fetal tissue consistently exhibited greater transcriptional variability, broader pathway engagement, and stronger activation of stress-related signaling compared to placental tissue, suggesting differential regulatory responses at the maternal-fetal interface under diabetic conditions.

## Discussion

Diabetic pregnancies present significant risks to both maternal and fetal development, with effects emerging early in embryogenesis. The maternal-fetal interface plays a central role in mediating these effects. However, the extent to which placental and fetal tissues differentially respond to diabetic stress is not fully understood. Previous studies have shown that maternal diabetes can alter placental development, including changes in placental size, trophoblast proliferation, and fetal stress responses [23–24], suggesting that structural and functional adaptations at the interface may influence downstream fetal outcomes. In this study, a dual-dataset bioinformatic approach was used to compare transcriptional responses in placental/decidual tissue and fetal tissue under diabetic conditions, providing a systems-level view of compartment-specific responses.

Analysis of placental/decidual tissue revealed highly constrained differential-expression at the probe level where only two probes reached BH-adjusted *p* < 0.05. Only one probe, *Atp1a3*, met both BH-adjusted *p* < 0.05 and |log_2_FC| > 1 criterion. Due to the limited number of threshold-defined DEGs, rank-based GSEA was used to evaluate pathway-level changes across placental/decidual gene-ranking distribution. GSEA analysis identified prominent negative enrichment of biological processes associated with ribosomal small subunit biogenesis, ribosome biogenesis, ribonucleoprotein complex biogenesis, RNA splicing, rRNA processing, and mRNA processing. KEGG GSEA similarly identified negative enrichment of the spliceosome, ribosome, cell cycle, DNA replication, and related pathways. These findings suggest that the comparatively modest differential expression observed at the individual level coexists with coordinated shifts across broader gene processes in RNA processing, translation, and cellular proliferation. Limited numbers of threshold-defined DEGs should therefore not be interpreted as an absence of transcriptional response within the maternal-fetal interface.

In contrast, fetal tissue exhibited broader and more variable transcriptional changes, as evidenced by wider expression distributions and more active pathway patterns. With 2,306 probes meeting the combined BH-adjusted *p* < 0.05 and |log_2_FC| > 1 criteria, corrected KEGG over-representation analysis identified 62 significantly enriched pathway, including pathways associated with axon guidance, Wnt signaling, focal adhesion, Hippo signaling, cell adhesion, and intracellular signaling. MAPK and PI3K-AKT signaling were also significantly enriched among fetal DEGS, consistent with widespread transcriptional alterations observed across components of these pathways in Pathview visualizations. MAPK signaling has an established role in cellular proliferation, differentiation, stress responses, and inflammatory signaling [27–28]. PI3K-AKT signaling contributes to growth, survival, and metabolic regulation. The broader distribution of fetal transcript-level alterations across these signaling networks is therefore consistent with disruption of developmental and stress-responsive processes under diabetic conditions. However, the direction of individual mapped transcript changes should not be interpreted as direct evidence of pathway activation or inhibition.

Pathway mapping further identified differences in the distribution of transcript-level changes across the KEGG Pathways of neurodegeneration – multiple disease pathway (mmu05022). Placental/decidual tissue exhibited comparatively localized changes, whereas fetal tissue showed much broader transcription-level changes including mitochondrial function, protein processing, and cellular stress. shared signatures of mitochondrial dysfunction and protein processing disruption in both placental and fetal tissues, with greater signal intensity observed in the fetal dataset. This pattern is consistent with increased susceptibility of the fetal compartment to metabolic stress. The patterns illustrated are interpreted as further descriptive pathways rather than evidence of neurodegeneration pathway activation.

Collectively, these findings are consistent with a model in which the maternal-fetal interface may partially modulate the effects of maternal diabetes, while the fetal compartment exhibits a more extensive and stress-responsive transcriptional profile. However, these findings do not directly demonstrate buffering because the datasets were independently generated and differ in various aspects such as composition, sample number, and pooling. These compartment-specific patterns are consistent with the Developmental Origins of Health and Disease (DOHaD) framework, in which suboptimal intrauterine signaling environments by the placenta may influence the developing fetus to long term metabolic and neurodevelopmental risk. Importantly, several of the pathway-level alterations identified in the present study are consistent with a growing body of evidence describing the molecular mechanisms underlying diabetic pregnancy. Recent studies have highlighted dysregulation of PI3K-AKT and MAPK signaling, inflammatory signaling, extracellular matrix remodeling, and mitochondrial dysfunction as key contributors to placental dysfunction and altered fetal development associated with maternal diabetes [23, 29–30]. Although an independent transcriptomic dataset containing comparable placental and fetal tissues was not available for direct validation, the concordance of the identified pathways with established biological mechanisms provides additional support for the transcriptomic patterns observed in the study.

Several limitations should be considered. First, this study was based on publicly available microarray datasets with relatively small sample sizes, particularly in the fetal datasets, which may have reduced statistical power to detect subtle expression changes. Second, the placental samples consisted of mixed placental and decidual tissue, and the fetal samples were generated from pooled embryos, limiting resolution of cell type-specific and individual-level responses. The marked differential expression of the decidual-associated transcript *Prl8a2* in the fetal dataset, together with expression changes observed in Supplementary Table S1C indicates that differential carry-over of maternal-interface tissue cannot be excluded. These findings do not establish tissue contamination but warrant caution with interpreting maternal-interface markers. Third, the placental/decidua dataset exhibited relatively modest transcriptional changes, with few genes meeting stringent differential expression criteria (BH-adjusted *p*-value < 0.05 and |log_2_FC| > 1). Accordingly, selected visualizations, including the placental heatmap, were based on the highest-ranked genes by BH-adjusted *p*-value rather than exclusively on statistically significant differentially expressed genes. Fourth, pathway enrichment and Pathview analyses are dependent on existing pathway annotations and provide associative rather than causal interpretations. Finally, the findings are computational and were not validated experimentally. Despite these limitations, the use of complementary placental and fetal datasets and a consistent bioinformatic workflow provides a robust framework for characterizing compartment-specific transcriptional responses to maternal diabetic stress.

These results highlight the value of integrative transcriptomic approaches in dissecting compartment-specific responses within the maternal-fetal interface. Future studies employing single-cell RNA sequencing and cell-type-specific analyses will be necessary to deconvolve the heterogeneous transcriptional contributions of individual trophoblast, decidual, and fetal cell populations, and to determine whether the transcriptional and pathway-level alterations identified here are causally linked to adverse developmental outcomes in diabetic pregnancies.

## Supplementary Information

Below is the link to the electronic supplementary material.

Supplementary Material 1

Supplementary Material 2

Supplementary Material 3

## Data Availability

No datasets were generated or analysed during the current study.
