# Supplementary material for: A bioinformatic approach to investigate placental and fetal gene expression during diabetic stress in pregnancy
Source: In Silico Pharmacol. 2026 Oct 23;14(3):267. doi: 10.1007/s40203-026-00778-2 (PMC13694575; doi:10.1007/s40203-026-00778-2)
Supplement: Supplementary file 1 [file 40203_2026_778_MOESM3_ESM.docx]

**Supplemental Figures**

**Figure S1. (A) Boxplots of gene expression values for placental/decidua samples before and after RMA normalization. Raw expression intensities (left) and log_2_-transformed, normalized values (right) are shown across samples; control samples are in blue and diabetic samples are in red. (B)** **MA plot of differential gene expression in placental/decidua tissue (diabetic vs. control). The x-axis shows average log2 expression (A value) and the y-axis shows log_2_ fold change (M value); each point represents a probe.**

**Figure S2. Cross-dataset comparison and quality assessment of differential expression. (A) Scatter plot of gene-level log_2_ fold changes with a fitted regression line. (B) The x-axis shows average log_2_ expression and the y-axis shows log_2_ fold change; each point represents a probe. (C) MA plots for selected arrays showing M (log-ratio) versus A (mean intensity). Each panel represents an array, with the x-axis showing average log intensity and the y-axis showing log-ratio.**

**Figure S3. Quality control assessment of placental/decidua and fetal microarray datasets. (A) RNA degradation plot showing probe intensity from 5’ to 3’ across samples. The x-axis represents probe position and the y-axis represents mean intensity; each line corresponds to a sample, with placental/decidua in red and fetal tissue in blue. (B) Density distributions of RMA-normalized gene expression values for placental/decidua and fetal tissues. Each curve represents a sample, with placental/decidua in red and fetal tissue in blue.**
